# Interventions for loneliness in older adults: a systematic review of reviews

**DOI:** 10.3389/fpubh.2024.1427605

**Published:** 2024-07-18

**Authors:** Uday Patil, Kathryn L. Braun

**Affiliations:** Office of Public Health Studies, Thompson School of Social Work & Public Health, University of Hawaiʻi at Mānoa, Honolulu, HI, United States

**Keywords:** aging, older adults, loneliness, social isolation, systematic review

## Abstract

Loneliness in older persons is a major risk factor for adverse health outcomes. Before the COVID-19 pandemic led to unprecedented isolation and hampered programs aimed at preventing or reducing loneliness, many interventions were developed and evaluated. However, previous reviews provide limited or conflicting summaries of intervention effectiveness. This systematic review aimed to assess previous review quality and bias, as well as to summarize key findings into an overarching narrative on intervention efficacy. The authors searched nine electronic databases and indices to identify systematic reviews of interventions to reduce loneliness in older people prior to the COVID-19 pandemic; 6,925 records were found initially. Of these, 19 reviews met inclusion criteria; these encompassed 101 unique primary intervention studies that varied in research design, sample size, intervention setting, and measures of loneliness across 21 nations. While 42% of reviews had minimal risk of bias, only 8% of primary studies appraised similarly. Among the 101 unique articles reviewed, 63% of tested interventions were deemed by article author(s) as effective or partially effective. Generally, interventions that included animals, psychological therapies, and skill-building activities were more successful than interventions focused on social facilitation or health promotion. However, interventions that targeted multiple objectives aimed at reducing loneliness (e.g., improving social skills, enhancing social support, increasing social opportunities, and changing maladaptive social cognition) were more effective than single-objective interventions. Future programs should incorporate multiple approaches, and these interventions should be rigorously tested.

## 1 Introduction

Reported prevalence of loneliness among older adults varies widely, with estimates from 7 to 63%, while many reports estimate a point prevalence around 20% (1–14). Incidence may be increasing throughout the world (1, 15–17). Some explanations for the increases in rates of loneliness are associated with increased longevity, greater years lived with disability, and degradation of social support over time (4, 18–21). An increase in single living and delayed marriage, along with a decrease in fertility rates and ability to spend time with loved ones due to delayed retirement, may also play significant roles (14, 19, 21–26). In the early 2020s, the COVID pandemic increased social isolation for all, which likely increased prevalence of loneliness among older adults.

Although the terms *loneliness* and *social isolation* have been used interchangeably, they are different constructs. Loneliness is an unwelcomed feeling of being removed from people and communities (3, 9, 16, 20, 27, 28). Social isolation refers to an objective lack of integration with others who would otherwise supply structural or functional social support. While analytic studies show an overlap of the terms as resulting in similar negative health consequences in older people (2, 8, 10–13, 17, 19, 29–34), the concepts are distinct (2, 8, 16, 17, 30, 31, 35–38). Moreover, the presence of one does not necessitate the presence of the other (10, 17, 39). This review spotlights loneliness only, as it is unequivocally unwanted, whereas some older adults may seek out social isolation.

Loneliness is commonly identified as a risk factor for adverse health outcomes, such as mental illness, cardiovascular disease, and early death (2, 5–7, 15, 16, 18, 23, 27, 29, 40, 41). Chronic loneliness is also associated with increased inpatient admissions, inpatient stay lengths, and emergency care visits (8, 22, 28). Many researchers compare the effects of chronic loneliness to those of cigarette smoking, sedentary lifestyle, obesity, and persistent hypertension (7, 15, 35, 42–44).

Researchers across disciplines have tested interventions to increase interpersonal engagement and combat loneliness (5, 8, 20, 28, 30, 36, 40, 41, 45, 46). Masi et al. categorized intervention objectives or aims into four area—improving social skills, enhancing social support, increasing social opportunities, and changing maladaptive social cognition (20, 35, 41). A thematic analysis by Gardiner et al. (30) described six main types of interventions: social facilitation, psychological therapies, health and social care provision, animal assistance activities, befriending programs, and leisure or skill-development activities.

The overall effectiveness of interventions is difficult to summarize. Numerous narrative and meta-analytic reviews have been published, but many focus on one type of intervention, including a review of reviews by Chipps et al. focused on information-community technology (ICT) interventions (47). Overall, the reviews provide inconsistent or conflicting summaries regarding effectiveness of individual approaches or types of approaches to combat loneliness (12, 13, 15). Also, while review authors have assessed the quality of the included studies, there has been limited reflection of quality of these reviews.

Thus, the purpose of this systematic review is to synthesize previously completed reviews. This overview is unique in that it focuses only on loneliness as an outcome. Moreover, it fills important research gaps by assessing the quality of each review article and summarizing key findings and data of previous reviews into a comprehensive narrative on intervention effectiveness.

## 2 Methods

The Preferred Reporting Items for Systematic Reviews and Meta-Analysis for Protocols (PRISMA-P) 2015 guidelines (48) were followed, but the protocol was unregistered.

### 2.1 Search methods

Under the guidance of a medical information specialist, search terms in the five PICOS categories were selected for Population (older adults, as defined by authors), Interventions to reduce loneliness, Comparator (any), Outcomes (loneliness), and Study design (systematic review). The authors tailored queries with associated controlled vocabulary per database (Appendix A). Nine electronic databases and indices were searched for systematic reviews written between January 1970 and July 2020. The authors investigated dissertations and gray literature for qualified refereed reviews published elsewhere. Upon recommendation of subject experts, the authors hand-searched *The Gerontologist* and *The Journals of Gerontology*. Citation tracking of included reviews discovered supplementary reviews to aid in narrative development.

### 2.2 Inclusion and exclusion criteria

Reviews must have summarized finding from the testing of interventions to alleviate loneliness as a primary or secondary goal among older adults (49). Reviews must have been peer-reviewed and systematic and presented quantitative or qualitative evidence detailing the effectiveness of interventions to prevent or reduce loneliness. The authors included reviews that examined interventions targeting corollary constructs, like social isolation and social participation, if one or more embedded studies aimed to reduce loneliness.

### 2.3 Article selection

After citations were found using the search strategy above, duplicates were removed. The Zotero 5 software suite was used to collect, manage, and cite sources (50). The authors identified prospective reviews from searches by scanning titles, then abstracts, and finally, full-text articles. Consensus was used to resolve eligibility concerns. The authors extracted review information in accordance with the Cochrane Effective Practice and Organization of Care Group (EPOC) using a modified form for systematic reviews of reviews (51, 52).

### 2.4 Categorization of interventions

Interventions were categorized by the authors into one of the four intervention objectives or aims identified by Masi et al.*—*improving social skills, enhancing social support, increasing social opportunities, and changing maladaptive social cognition (46). They also were categorized by type of intervention as outlined by Gardiner et al.—social facilitation, psychological therapies, health and social care provision, animal assistance activities, befriending programs, and leisure or skill development activities (23).

### 2.5 Risk of bias analysis

Systematic reviews were assessed for risk of bias via *A MeaSurement Tool to Assess systematic Reviews* (AMSTAR 2) (53). Appraisal of critical and non-critical items (as defined by the tool) established summary ratings of High, Moderate, Low, and Critically Low. Due to the heterogeneity of approaches, interventions, populations, and outcomes, the authors did not conduct a meta-analysis of underlying studies (51, 54).

## 3 Results

**Figure 1:**
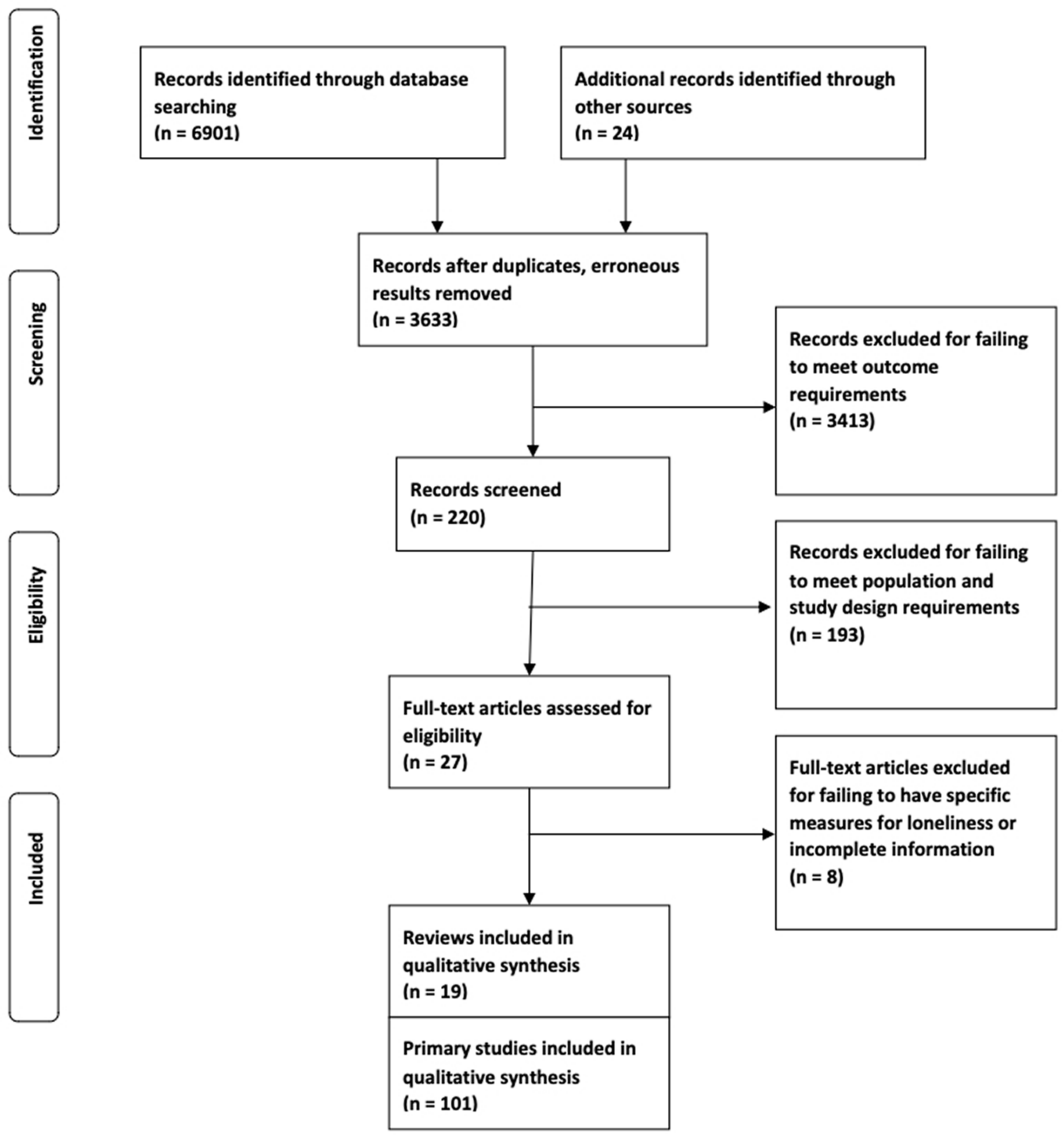
PRISMA flowchart of screening and selection.

A search conducted in August 2020 yielded 6,901 records, and another 24 records were identified through citation chasing. Of the 6,925 total records, titles of 6,705 clearly indicated that they were not relevant to this review and were eliminated. The abstracts of the 220 remaining records were screened, and 193 more were excluded. The remaining 27 reviews were read in full. Eight of these held incomplete information or failed to include explicit measures for loneliness. Thus, 19 systematic reviews were included. These encompassed 212 primary research studies, of which 101 (47%) were unique (Figure 1).

### 3.1 Characteristics

The characteristics of the 19 reviews are shown in Table 1. The median year of publication is 2016, with only one review published prior to 2010. Of the 19, two systematic reviews provided meta-analyses (67, 68), and one was the aforementioned review of systematic reviews of ICT of interventions (47). Eight reviews (42%) were general in nature (30, 40, 41, 69–73), while seven (37%) focused on technological interventions (11, 47, 68, 74–77), and four (21%) focused on physical or mental health promotion activities (8, 67, 78, 79).

**Table 1:** Review summary.

Author and Ref.	Focus of review	Number of articles by focus	Age of study participants	Gender of participants	Intervention setting	n	Measure of loneliness	% of effective loneliness interventions
Baker et al., 2018 (11)	Systematic review of assistive technology interventions, 2000–2016	36 articles testing interventions using assistive technologies; 2 met inclusion criteria.	Partially disclosed; older persons	Undisclosed	Undisclosed	8–388	Undisclosed	50%
Bemelmans et al. 2012 (55)	Systematic review of interventions using socially assistive robots, Earliest - 2009	17 articles testing interventions using socially assistive robots 2 met inclusion criteria.	Partially disclosed; older persons	Partially disclosed; mostly female	Residential care facilities; international	5–26	UCLA LS; AOKLS	100%
Bermeja et al., 2018 (56)	Systematic review, 2000–2016	11 articles testing different interventions (animal-assisted, videoconferencing, horticulture workshops, reminiscence therapy, humor therapy, cognitive interventions) to reduce loneliness 11 met inclusion criteria.	60–98	Partially disclosed; mostly male	Residential care facilities; international	10–396	Philadelphia Vital Satisfaction Scale, UCLA LS, SESLA-Spanish, Emotional–Social Loneliness Inventory, ESTE Loneliness Scale, dJG	91%
Cattan et al., 2005 (8)	Systematic review of health promotion interventions, 1970–2002	30 articles testing health promotion interventions 8 met inclusion criteria.	Partially disclosed; mean age 65+	Partially disclosed; mixed	Residential care facilities, community, private homes; international	23–1,555	UCLA LS, dJG, 9 proprietary scales	50%
Chen et al., 2016 (57)	Systematic review of Information communication technology interventions 2002–2015	25 articles testing information communication technology interventions 18 met inclusion criteria.	Partially disclosed; mean age 60+, 55–93	Partially disclosed; mixed	Residential care facilities, community; international	8–5,203	dJG, proprietary scales, UCLA LS, Hughes Loneliness Scale, SELSA	89%
Chipps et al., 2017 (47)	Systematic review of systematic reviews testing information communication technology interventions, 2000–2017	12 and 22 studies testing information communication technology interventions 20 met inclusion criteria.	Partially disclosed; older persons, 55+	Mixed	Residential care facilities, community; international	3–236	UCLA LS, dJG	60%
Cohen-Mansfield et al., 2015 (17)	Systematic review, 1996–2011	34 articles testing different interventions (shared activities, educational events, technology-based aids) to reduce loneliness 29 met inclusion criteria.	50+, mean age 60+	Partially disclosed; mostly female	Residential care facilities, community, private homes; international	9–708	UCLA LS; PGCMS; dJG; proprietary 2-item instrument; proprietary 1-item self-report	59%
Dickens et al., 2011 (18)	Systematic review of group and one-on-one service provision interventions, Earliest – 2009	32 articles testing group and one-to-one, service provision interventions 16 met inclusion criteria.	Undisclosed; older persons	Undisclosed	Residential care facilities, community; international	23–741	UCLA LS, dJG, modified UCLA LS	31%
Elias et al., 2015 (58)	Systematic review of group reminiscence therapy, 2002–2014	8 articles testing group reminiscence therapy 1 article targeting loneliness as an outcome	Undisclosed; older persons	100% male	Residential care facilities; Taiwan	92	UCLA LS	100%
Franck et al., 2016 (59)	Systematic review, 2009–2013	34 articles testing different interventions (reminiscence therapy, active gaming, indoor gardening, radio program) 4 met inclusion criteria.	Partially disclosed; mostly 60+, one study mean age 82	Partially disclosed; mostly female	Residential care facilities; international, urban	24–130	UCLA LS; Victor	75%
Gardiner et al., 2018 (23)	Systematic review, integrative review, 2003–2016	38 articles testing different interventions (social facilitation, psychological therapies, health and social care provision, leisure/skill development, befriending intervention) 31 met inclusion criteria.	Partially disclosed; 52+, mean age 60+	Undisclosed	Residential care facilities, community, private homes; international	Partially disclosed; 4–817	UCLA LS, dJG, proprietary interview questions, proprietary questionnaire, US Health and Retirement Study loneliness items	71%
Hagan et al., 2014 (60)	Systematic review 2000–2012	17 articles testing different interventions (group, one-to-one mentoring, recent technology interventions) to reduce loneliness 14 met inclusion criteria.	Partially disclosed; mean age 65+	Partially disclosed; mostly female	47% community, 41% residential care facility, 12% day center; international	26–1,217	UCLA LS; dJG; proprietary survey; well-being scales	36%
Kachouie et al., 2014 (61)	Systematic review of socially assistive robots Earliest – 2012	38 articles testing socially assistive robots 2 met inclusion criteria.	Partially disclosed; older persons, mean age 70.8 in one study	Partially disclosed; mostly female	Residential care facilities; international	6–38	UCLA LS, AOS Loneliness Scale	100%
Li et al., 2018 (62)	Systematic review, bibliometric analysis Earliest – 2017	10 articles testing exercise and digital games. 3 met inclusion criteria.	Partially disclosed; 55+, mean age 60+	Undisclosed	Day centers, community; international	35–113	UCLA LS	100%
Poscia et al., 2018 (63)	Systematic review, update 2011–2015	20 articles testing interventions to reduce loneliness and social isolation 12 met inclusion criteria.	Partially disclosed; mean age 60+	Partially disclosed; mostly female	Residential care facilities, community, private homes; international	13–858	AOKLS, dJG, Italian version of Loneliness Scale (ILS), dJG, Loneliness Literacy Scale, UCLA LS,	58%
Pu et al., 2018 (64)	Systematic review, meta-analysis of socially assistive robots Earliest – 2017	11 articles testing socially assistive robots 2 met inclusion criteria.	Partially disclosed; 55–100	Partially disclosed; mostly female	Residential care facility, hospital; international	38–40	UCLA LS	100%
Shvedko et al., 2018 (65)	Systematic review, meta-analysis of physical activity interventions Earliest – 2017	23 articles testing physical activity interventions 3 met inclusion criteria.	Mean ages, 77.3 (7.4), 70.8 (5.2), 78.4 (6.6)	Mostly female	Community, day centers; international	41–708	One-item question, UCLA LS, and dJG	33%
Sims-Gould et al., 2017 (57)	Systematic review of reablement, reactivation, rehabilitation, and restorative (4R) interventions Earliest – 2016	15 articles testing reablement, reactivation, rehabilitation, and restorative (4R) interventions 1 article met inclusion criteria.	Mean age 82	75.3% female	Private homes	88	dJG	100%
Snowden et al., 2014 (66)	Systematic review of social support, strength and resistance training Earliest – 2012	148 articles testing social support, strength and resistance training 2 met inclusion criteria.	Undisclosed; older persons	Undisclosed	Undisclosed	32–313	Undisclosed	0%

Ref., Reference; AOKLS, Ando Osada and Kodama Loneliness Scale; dJG, De Jong Gierveld Loneliness Scale; PGCMS, Philadelphia Geriatric Center Morale Scale, Lonely Dissatisfaction Subscale; SELSA, Social and Emotional Loneliness Scale for Adults; UCLA LS, University of California Los Angeles Loneliness Scale; Victor, Victor single-item scale.

Only three of the reviews limited their study to articles expressly testing intervention impact on loneliness (17, 56, 60), while the other 16 reviews included a subset of articles testing an intervention’s impact on loneliness. For example, Elias et al. reviewed eight articles testing the impact of group reminiscence therapy on alleviating depression, anxiety, and loneliness, with only one article targeting loneliness as an outcome (58).

**Figure 2:**
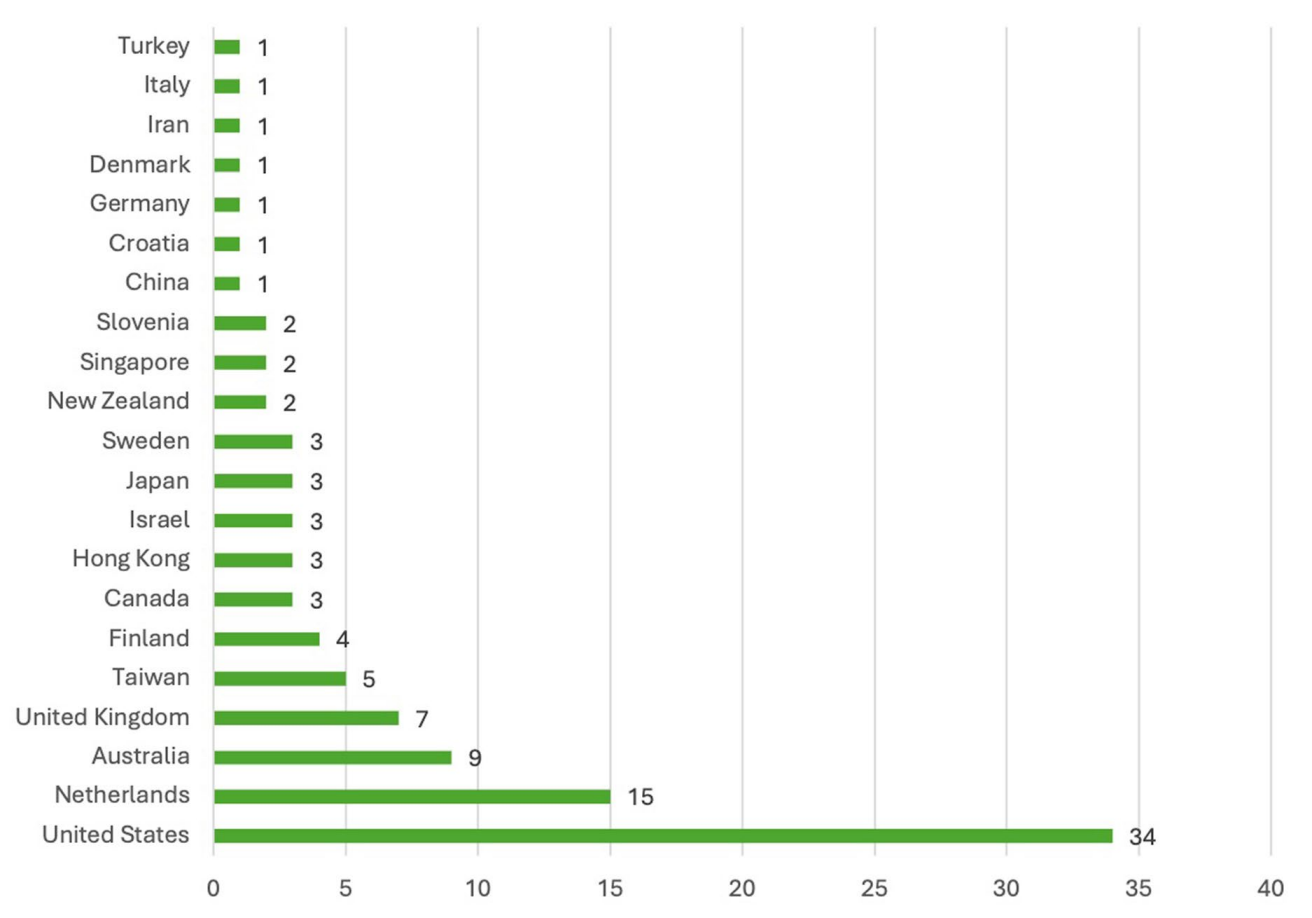
Geographic distribution of primary studies.

Characteristics of the 101 primary studies (including only one of the eight in the Elias et al. review) are shown in Table 2. About half (52) of the 101 primary studies were published after 2010. While 69 (68%) of the articles were included in only one of the 19 review articles, 42 were included in two or more of the review articles. Overall, studies sampled populations from 21 nations (Figure 2); including 35 in Europe and the United Kingdom, 34 in the United States, 14 in Asia, 11 in Australia/New Zealand, five in Middle Eastern countries, and three in Canada.

**TABLE 2:** Intervention summary.

	Review reference	Study reference	Quality	Intervention activities	Effect	Intervention objective or aim per Masi (46)			
						Improving social skills	Enhancing social support	Increasing social opportunities	Changing maladaptive soc. cognition
Multi-category program	(40)	(59)	M-H	Multifaceted activity intervention	**●**	√	√	√	√
	(30, 40, 41, 67)	(80)	M	Multifaceted health intervention	**○**		√	√	
	(41)	(81)	M	Wellness education group	**●**	√	√	√	√
	(79)	(82)	H	Dutch Geriatric Intervention Program	**●**		√	√	√
	(30, 70)	(83)	L-M	CareTV video network support	**●**		√	√	
	(8)	(84)	H	Social activation and support programs	**●**	√	√	√	√
Psychological therapy	(40)	(85)	L-M	Telephone support group	**●**		√	√	
	(8, 40)	(86)	L-M	Support group, peer leadership	**●**	√	√	√	
	(30, 40, 41, 72)	(87)	M-H	Psychosocial group intervention	**○**	√	√	√	√
	(30, 40, 69)	(60)	M	Humor therapy	**●**	√	√	√	√
	(70)	(88)	L	Reminiscence therapy	**●**	√	√	√	√
	(70)	(89)	L	Psychoeducation, social activation	**○**	√	√	√	√
	(40, 41, 72)	(66)	L-M	Self-management skills course	**●**	√	√	√	√
	(41)	(90)	M	Coping education	**○**		√	√	√
	(8)	(91)	H	Social skills education	**●**		√	√	
	(69, 71, 78)	(92)	M	Reminiscence group	**●**	√	√	√	√
	(30)	(93)	M	Reminiscence therapy	**●**	√	√	√	√
	(40)	(94)	L	Social networks and health management	**●**	√	√	√	
	(40)	(95)	L-M	Psychosocial skills with caregivers	**●**	√	√	√	
	(30, 72)	(96)	M	Mindfulness stress reduction	**●**	√	√		√
	(41)	(97)	L	Telephone support group	**●**	√	√	√	
	(8)	(98)	H	Counseling and self-help training	**●**	√	√	√	√
	(30, 40, 41, 69, 72)	(99)	M	Cognitive therapy	**○**	√	√		√
Animal-assisted Intervention	(69)	(100)	L	Dog companionship	**●**	√	√		
	(47, 75, 77)	(63)	M	Robotic pet (AIBO) companionship	**●**	√	√		
	(68, 70)	(101)	L-M	Robot (PARO) companionship	**●**	√	√	√	
	(30, 47, 68, 72, 75, 77)	(102)	M	Pet and robotic pet (AIBO) companionship	**●**	√	√		
	(40, 69)	(103)	M	Animal-assisted therapy	**●**	√	√		
	(30)	(65)	M	Animal-assisted therapy, socialization	**●**	√	√	√	
	(30)	(64)	L-M	Pet ownership	**●**	√	√		
	(76)	(62)	L	Virtual pet (Gerijoy)	**●**		√	√	
Health Promotio**n**	(8, 40)	(104)	M-H	Hearing aids	**○**		√	√	
	(8)	(105)	H	Health, social home visits	**○**		√	√	
	(69)	(106)	M	Exercise program	**●**		√	√	
	(67)	(55)	M	Tai chi qigong	**●**		√	√	√
	(30, 70)	(57)	M	Health promotion (Pender)	**●**	√	√	√	√
	(40)	(107)	M-L	Occupational therapy, assistive devices	**○**		√	√	
	(8)	(58)	H	Exercise program, health education	**●**			√	
	(8)	(108)	H	Educational home visits	**○**		√	√	
	(76)	(109)	L-M	Telehealth system	**●**	√	√	√	
	(8)	(110)	M-H	Health, social home visits	**○**		√		
	(67)	(111)	M	Walking program	**○**		√	√	
	(40, 47)	(112)	M	AI exercise advisor	**○**		√	√	
	(73)	(113)	M	Strength training video	**○**			√	
	(40)	(114)	M	Exercise program	**●**		√	√	
Social facilitation	(30, 70)	(115)	L	Community services integration, psychoeducation	**○**	√	√	√	
	(70)	(116)	L	Singing sessions	**○**			√	
	(72)	(117)	M	Day center services	**○**		√	√	
	(30, 70)	(56)	L	Community networking, psychoeducation	**●**	√	√	√	√
	(30, 40, 41, 72)	(118)	M	Friendship enrichment training	**○**	√	√	√	
	(41)	(119)	L	Educational friendship program [Study 1]	**●**	√	√	√	
	(30, 40)	(119)	M	Educational friendship program [Study 2]	**●**	√	√	√	
	(30, 76)	(120)	L-M	Telephone befriending program	**●**		√	√	
	(72)	(121)	M	Community mentoring	**○**	√	√	√	
	(30)	(122)	M	Friendship programs	**●**	√	√	√	
	(30)	(123)	M	Telephone befriending program	**●**		√	√	
	(30, 41)	(124)	L	Pets, plans, children exposure	**○**	√		√	
	(30, 72)	(125)	M	Companionship program	**○**		√	√	
	(41, 73)	(126)	L-M	Telephone social support	**○**		√	√	
	(47)	(127)	M	AI conversational agent	**○**		√	√	
	(40, 41)	(128)	L	Foster grandparent program	**○**	√	√	√	
	(72)	(129)	M	Multiethnic community engagement	**○**	√	√	√	
Leisure or skill development	(30)	(130)	L-M	Social networking, internet use	**●**		√	√	
	(30, 70)	(115)	L	Art, fitness leisure program	**○**		√	√	
	(30, 70)	(115)	L	Cultural activities	**●**	√	√	√	
	(70)	(131)	L	Lifestyle engagement	**○**	√	√	√	
	(30)	(132)	M	Leisure activities	**●**		√	√	
	(76)	(133)	L	Internet use	**●**		√	√	
	(40, 71)	(134)	M	Radio program	**○**	√			
	(76)	(135)	L	ICT, mobile phone use	**●**		√	√	
	(30, 47, 76)	(136)	L-M	ICT training/use	**●**		√	√	
	(76)	(137)	L	ICT training/use	**●**		√	√	
	(40, 47, 76)	(138)	M	Community ICT training	**●**		√	√	
	(47, 76)	(139)	L-M	Social networking	**○**	√	√	√	
	(30, 40, 41, 47, 76)	(140)	L-M	ICT training/use	**●**		√	√	
	(40, 41, 47)	(141)	M-H	ICT training/use	**○**		√	√	
	(30, 47)	(142)	L-M	ICT training/use	**○**		√	√	
	(70)	(143)	L	ICT training/use	**●**		√		
	(74)	(144)	M-H	Video gaming (Wii)	**●**		√	√	
	(74)	(145)	M	Video gaming (Kinect)	**●**		√	√	
	(11)	(146)	M	Social networking	**○**	√	√	√	
	(76)	(147)	L	Videoconferencing	**●**		√	√	
	(47)	(148)	L-M	ICT training/use	**●**		√	√	
	(69)	(149)	L	Horticulture	**●**			√	
	(30, 40, 69, 71)	(150)	M	Horticulture	**●**			√	
	(30, 40, 47, 69, 76)	(151)	M	Videoconferencing	**●**		√	√	
	(40, 47, 69, 72, 76)	(152)	M	Videoconferencing	**●**		√	√	
	(72)	(153)	M	Video gaming (Wii)	**○**		√	√	
	(47)	(154)	L-M	Social networking	**○**	√	√	√	
	(30, 69)	(155)	M	Horticulture	**●**			√	
	(76)	(156)	M	ICT training/use	**●**		√	√	
	(40)	(157)	M	Choral participation	**●**			√	
	(11, 47, 76)	(158)	M	ICT training/use	**●**		√	√	
	(30, 47, 76)	(159)	L-M	ICT training/use	**●**		√	√	
	(47)	(160)	L-M	ICT training/use	**●**			√	
	(47, 71, 72, 74, 76)	(161)	H	Video gaming (Wii), TV use	**●**		√	√	
	(72)	(162)	M	Videoconferencing	**○**		√	√	
	(40)	(163)	L	Electronic pen pals	**●**	√	√	√	
	(40, 41, 47, 76)	(164)	M-H	ICT training/use	**○**		√	√	
	(40, 41)	(165)	L	ICT training/use	**○**		√	√	
	(47)	(166)	M-H	ICT training/use	**○**	√	√	√	

*●*, Effective or Partially Effective; ○, Not Effective or Inconclusive; √, Present; Quality L, Low, M, Moderate, H, High; ICT, Internet & Information Communication Technology.

Studies tested interventions using assorted designs, including controlled trial, clustered controlled trial, quasi-experimental design, pre-experimental (before-and-after) design, cross-sectional, and mixed-method types. Samples ranged from 3 to 5,203 subjects. Interventions occurred in residential care facilities, community day centers, and private homes. While some subjects were as young as 52 years old, the mean age of subjects in each study was above 60 years. Only some studies disclosed full gender characteristics. Six different measures were used across the 101 studies to measure loneliness.

Intervention types per Gardiner et al. (first column), activities (fifth column), and objectives per Masi et al. (last four columns) also are shown in Table 2. In terms of intervention objectives, only 10 of the 101 studies had a single objective, while 50 had two, 28 had 3, and 13 aimed to target all 4 areas. Thus, 91 of the 101 studies had an objective to enhance social support, 91 aimed to increase social opportunities, 46 strove to improve social skills, and 18 were designed to change maladaptive social cognition.

In terms of intervention type, 39 of the 101 studies tested interventions offering leisure or skill-building activities, 17 evaluated psychological therapies, 17 tested social facilitation interventions, 14 evaluated health promotion interventions, eight (8%) gaged animal-assisted interventions, and six (6%) assessed multi-category programs. While 88% of the psychological therapies and 67% of the multi-category interventions had three or more intervention objectives (e.g., to enhance social support, improve social skills and change maladaptive behavior), health promotion programs and leisure and skill-building activities tended to have fewer intervention objectives.

### 3.2 Effectiveness

Table 1 recaps included systematic reviews. Review authors gaged interventions to be mostly of mixed effectiveness when aiming to reduce loneliness in older persons. Most reviews found some support for both group and individual-targeted interventions; however, at least one general and one health intervention review found group interventions to be more effective (8, 41) and at least one general review found the converse (70).

Six (75%) of eight general reviews obtained mixed results, while one (13%) concluded interventions to be mostly effective (30), and one (13%) avoided a conclusion due to insufficient evidence (73). Regarding reviews appraising technological interventions, five (71%) of seven reviews summarized this type to be mostly effective, while one (14%) review found mixed efficacy for some assistive technology interventions such as social networking services (11), and one (14%) review could not provide a conclusive evaluation due to the limitations of underlying studies (47). Reviews focused on physical and mental health promotions stated ambiguous results of their effectiveness: one (25%) of four reviews provided evidence that group reminiscence therapy approaches are effective (78), while two (50%) reviews found no overarching proof of programmatic efficacy (67, 79). One (25%) review by Cattan et al. relayed assorted results of interventions combatting loneliness (8).

Regarding intervention objective, researchers found 14 (78%) of 18 interventions focused on changing maladaptive social cognition, 31 (67%) of 46 on improving social skills, 59 (65%) of 91 on enhancing social support, and 57 (63%) of 91 on increasing social opportunities to be effective or partially effective. Five (50%) of 10 of uni-objective intervention, 32 (64%) of 50 bi-objective interventions, 16 (57%) of 28 of tri-objective interventions, and 11 (85%) of 13 complete, quad-objective studies were effective or partially effective.

### 3.3 Quality

Table 3 details estimates of study quality of each systematic review. The authors appraised 8 (42%) of 19 reviews to be of high quality (8, 41, 67, 68, 71, 76, 78, 79), with another eight (42%) being of moderate-high quality. These reviews displayed a minimal risk of bias. Two reviews (11%) were assessed as of moderate quality, and one (5%) was deemed low-moderate quality. Every health promotion review was high-quality. In contrast, only two (29%) of seven reviews appraising technology-based interventions and two (25%) of eight general intervention reviews were of high quality.

**Table 3:** Review quality per AMSTAR 2 guidelines.

Author	Ref.	PICOS Criteria	Protocol Established	Study Justification	Search Strategy	Duplicate Selection	Duplicate Extraction	Exclusion List	Study Detail	Study Biases	Study Funding	MA: Effects	MA: Biases	Biases Discussion	Heterogeneity	MA: Publication Bias	Conflict of Interests	Quality
Baker et al.	(11)	⬤	⬤	⬤	◐	⬤	⬤	⬤	◐	⬤	⬤			⬤	⬤		⬤	Moderate–High
Bemelmans et al.	(75)	⬤	⬤	⬤	◐	⬤	⬤	⬤	⬤	⬤	⬤			⬤	⬤		⬤	Moderate
Bermeja et al.	(69)	⬤	⬤	⬤	◐	⬤	⬤	⬤	⬤	⬤	⬤			⬤	⬤		⬤	Moderate–High
Cattan et al.	(8)	⬤	⬤	⬤	◐	⬤	⬤	⬤	⬤	⬤	⬤			⬤	⬤		⬤	High
Chen et al.	(76)	⬤	⬤	⬤	◐	⬤	⬤	⬤	⬤	⬤	⬤			⬤	⬤		⬤	High
Chipps et al.	(47)	⬤	⬤	⬤	◐	⬤	⬤	⬤	⬤	⬤	⬤			⬤	⬤		⬤	Moderate–High
Cohen-Mansfield et al.	(40)	⬤	⬤	⬤	◐	⬤	⬤	⬤	⬤	◐	⬤			⬤	⬤		⬤	Moderate–High
Dickens et al.	(41)	⬤	⬤	⬤	◐	⬤	⬤	⬤	◐	⬤	⬤			⬤	⬤		⬤	High
Elias et al.	(78)	⬤	⬤	⬤	◐	⬤	⬤	⬤	◐	⬤	⬤			⬤	⬤		⬤	High
Franck et al.	(71)	⬤	⬤	⬤	◐	⬤	⬤	⬤	⬤	⬤	⬤			⬤	⬤		⬤	High
Gardiner et al.	(30)	⬤	⬤	⬤	◐	⬤	⬤	⬤	◐	⬤	⬤			⬤	⬤		⬤	Moderate–High
Hagan et al.	(72)	⬤	⬤	⬤	◐	⬤	⬤	⬤	⬤	⬤	⬤			⬤	⬤		⬤	Low–Moderate
Kachouie et al.	(77)	⬤	⬤	⬤	◐	⬤	⬤	⬤	⬤	⬤	⬤			⬤	⬤		⬤	Moderate–High
Li et al.	(74)	⬤	⬤	⬤	◐	⬤	⬤	⬤	◐	⬤	⬤			⬤	⬤		⬤	Moderate
Poscia et al.	(70)	⬤	⬤	⬤	◐	⬤	⬤	⬤	⬤	⬤	⬤			⬤	⬤		⬤	Moderate–High
Pu et al.	(68)	⬤	⬤	⬤	⬤	⬤	⬤	⬤	⬤	⬤	⬤	⬤	⬤	⬤	⬤	⬤	⬤	High
Shvedko et al.	(67)	⬤	⬤	⬤	⬤	⬤	⬤	⬤	⬤	⬤	⬤	⬤	⬤	⬤	⬤	⬤	⬤	High
Sims-Gould et al.	(79)	⬤	⬤	⬤	⬤	⬤	⬤	⬤	⬤	⬤	⬤			⬤	⬤		⬤	High
Snowden et al.	(73)	⬤	⬤	⬤	◐	⬤	⬤	⬤	⬤	⬤	⬤			⬤	⬤		⬤	Moderate–High

ID, Reference; MA, Meta-analysis; ⬤, Yes; ◐, Partial Yes; ⬤, N.

In accordance with the AMSTAR 2 guidelines (53), the authors accounted for the following three criteria when developing a summary of review quality. No reviews fully disclosed information regarding primary study funding per Item 10. Most reviews failed to provide a comprehensive list of excluded studies per Item 7. Only 24 studies (24%) employed randomized controlled trial (RCT) designs. Only two studies provided a meta-analysis (67, 68); hence, these were the only ones subject to Items 11, 12, and 15.

Table 2 also lists quality assessment, including grading criteria, for each of the 101 studies within the 19 reviews. The authors found only eight (8%) of the 101 studies to be of high quality (58, 82, 84, 91, 98, 105, 108, 161). Eight (8%) were between medium and high quality, 42 (42%) were of medium quality, 20 (20%) between low and medium quality, and 23 (23%) were of low quality. High-quality investigations were rare across intervention objectives, e.g., only two (4%) of 46 intervention that aimed to improve social skills, 7 (8%) of 91 interventions that aimed to enhance social support, 8 (9%) of the 91 that aimed to increase social opportunities, and 3 (17%) of 18 that aimed to change maladaptive social cognition to be of high quality.

Additionally, Table 2 lists the efficacy of each intervention, as noted by the reviews and studies themselves. Of the 101 underlying studies, primary investigators concluded 64 (63%) to be effective or partially effective. However, this varied by study designs, e.g., only 12 of the 24 programs tested through RCT were found to be effective. Irrespective of study methodology, all eight (100%) animal-assisted interventions, five (83%) of six multi-category programs, 13 (76%) of 17 psychological therapies, 26 (67%) of 39 leisure or skill-building activities, 6 (43%) of 14 health promotions, and 6 (35%) of 17 social facilitations were effective or partially effective.

## 4 Discussion

To the authors’ knowledge, this is the first systematic review of reviews of interventions to combat loneliness in older people. Nineteen systematic reviews amassed the findings of 101 unique studies of interventions. While 42% of the reviews were of the highest quality and contained minimal risk of bias, only 8% of primary studies were of the highest quality according to reviewers.

Regarding usefulness, the authors deducted that 63% of all interventions were effective or possibly effective at combatting loneliness. Multi-category interventions were above-par, along with programs featuring reminiscence therapies (88, 92, 93) and Mindfulness-Based Stress Reduction (96). All animal-assisted approaches were efficacious in combatting loneliness, including living (64, 65, 100, 102, 103), robotic (63, 101, 102), and virtual pet companionship (62). In addition, key findings support interventions with multiple objectives, as 85% of interventions with four objectives (improving social skills, enhancing social support, increasing social opportunities, and changing maladaptive social cognition) alleviated loneliness. The most successful single-objective interventions were those targeting maladaptive social cognition (55–57, 59, 60, 66, 81, 82, 84, 88, 92, 93, 96, 98), presumably to help lonely older adults develop more stable interpersonal relationships and perpetuate social opportunities. This finding is consistent with the hallmark meta-analysis by Masi et al. (35) on subjects of any age.

### 4.1 Limitations

Various considerations tempered the conclusions of this research. First, the authors limited the search to the pre-COVID years. Second, the included systematic reviews had differing foci and scopes, and this heterogeneity hindered comparisons across reviews. Many systematic reviews included were of moderate-high and high quality, but some displayed an elevated risk of bias (72, 75). Likewise, many of the studies testing a single intervention exhibited moderate-to-high risk of bias as a product of poor study design.

This systematic review of reviews compiled studies that utilized a variety of loneliness-related outcome measures. While some (i.e., UCLA Loneliness Scale, De Jong Gierveld Loneliness Scale) were well-tested with older people and psychometrically sound (61, 167–169), others were single-item measures or instruments of disputed reliability and validity (8). Also, this review provided a dichotomous summary statistic of effectiveness in its analyses, which reduced complex findings into manageable figures for easy comparison. Binning of interventions by intervention objective is a highly subjective task. Scholars should exercise caution when reducing constructs as complex as loneliness and social isolation into crude metrics, especially together, at the risk of misinterpreting primary study authors’ conclusions (29, 170).

### 4.2 Recommendations

Three findings stand out. First, allied health professions should develop broad interventions. A multi-objective approach aptly targets the multi-dimensional issue of loneliness (69, 76, 171, 172). Some participants of such interventions may find certain components useful, while other participants would find distinct parts worthwhile. Increasing the number of strategies can target the widest range of participants. This explains the above-average effectiveness ratings of integrated approaches to combating loneliness. The Dutch Geriatric Intervention Program (82) and Finnish psychosocial group rehabilitation intervention (59) are illustrative of this approach. Conclusions here are consistent with the best practices of robust health promotion initiatives targeting a variety of outcomes (173, 174).

Second, interventions should become more purpose-driven (67, 71) to stem the losses of identity many lonely older adults feel (78, 175). Shvedko et al. remarked that the theory of active engagement explains loneliness reduction through a productive lifestyle that generates a sense of purpose (67, 176). Effective programs provide more than aimless social opportunities (30, 132), and more than friendly health and social care visitations, as Cattan et al. found (8). Prime examples of purpose-driven approaches are horticulture-learning experiences (60, 149, 155) and fitness-improving “exergames” (144, 145, 161). The authors also observed specific, purposeful technology trainings to be effective in reducing loneliness, including programs utilizing mobile phones (135), electronic pen pals (163), and videoconferencing software (147, 151, 152).

Third, specific types of interventions proved to be more promising than others. Psychotherapeutic interventions utilized the highly effective strategy of modifying maladaptive social cognition—specifically engaging the theoretical mechanism of action noted by Cacioppo and others (5, 15). Animal-assisted interventions were helpful in providing purpose, delivering skills training, and increasing social opportunities for older people (62–65, 100–103), a finding that Banks et al. consistently espoused (65, 102, 103). Finally, technological interventions exhibited potential even as multiple reviews found inconclusive evidence (11, 47, 149). Chen et al. wrote “the older adults employment of [ICT] reduces their social isolation through the following mechanisms: connecting to the outside world, gaining social support, engaging in activities of interest, and boosting self-confidence” (76). Simple interventions, with little-to-no expert training or sharing were not effective (71), but approaches that demonstrated technology as a tool to encourage mobility, communication, or education exhibited high value (68, 74, 177).

Further studies of interventions to combat loneliness are needed. The authors request more individual or cluster RCTs to ensure a high-quality body of primary research not limited by risks of bias. Research scientists should heed the differences between social isolation and loneliness, lest phenomenological conclusions become confounded. Lastly, the authors concur with others who note plausible cultural moderators of intervention efficacy (8, 30, 40, 74, 75, 77) and encourage further examination of culture in perceptions of loneliness and ways to combat it.

## 5 Conclusion

The COVID-19 pandemic and associated quarantine orders further exacerbated the loneliness faced by many older adults (178). As health policies combatting loneliness quickly develop—like the national effort in the United Kingdom (179–181) or the health service company-led strategies in the United States (182–185)—researchers must begin to decipher years of equivocal findings and offer actionable recommendations. This report’s value lies in being the first systematic overview of the evidence base on loneliness interventions targeting older people in an attempt to help answer the question “What does an effective intervention look like?” Our findings suggest that interventions utilizing multiple strategies while incorporating purposeful activities are vital in disrupting loneliness and its deleterious effects in older adults.

## Data availability statement

The raw data supporting the conclusions of this article will be made available by the authors, without undue reservation.

## Author contributions

UP: Conceptualization, Data curation, Formal analysis, Investigation, Methodology, Software, Visualization, Writing – original draft, Writing – review & editing. KB: Conceptualization, Investigation, Methodology, Project administration, Resources, Supervision, Validation, Writing – review & editing.
