# Supplementary material for: Interventions for loneliness in older adults: a systematic review of reviews
Source: Front Public Health. 2024 Jul 18;12:1427605. doi: 10.3389/fpubh.2024.1427605 (PMC11291379; doi:10.3389/fpubh.2024.1427605)
Supplement: Supplementary file 1 [file Data_Sheet_1.docx]

## **Appendix A: Search Strategy**

| **Database** | **Query** | **Range** | **Results** |
| --- | --- | --- | --- |
| Academic Search Complete via EBSCOhost | older* OR elder* OR senior* OR geriatric OR aged OR “late life” OR “late onset” OR “old age” ) AND ( lonel* OR "social isolation" ) AND ( promot* OR prevent* OR support OR program* OR educat* OR reduc* OR minim* OR less* OR therap* OR interven* OR treat* ) AND ( review OR overview OR evaluat* OR discuss* OR “systematic review” or “meta-analysis” or “research synthesis” or “research integration” or “information synthesis” or “data synthesis” or “data extraction” | 1970 to present | 1128 |
| CINAHL via EBSCOhost | ( older* OR elder* OR senior* OR geriatric OR aged OR “late life” OR “late onset” OR “old age” ) AND ( lonel* OR "social isolation" ) AND ( promot* OR prevent* OR support OR program* OR educat* OR reduc* OR minim* OR less* OR therap* OR interven* OR treat* ) AND ( review OR overview OR evaluat* OR discuss* OR “systematic review” or “meta-analysis” or “research synthesis” or “research integration” or “information synthesis” or “data synthesis” or “data extraction” ) | 1970 to present | 1100 |
| Cochrane Database of Systematic Reviews via Cochrane Library | "social isolation" OR loneliness (ti, ab, kw) | All time | 19 |
| ERIC via ProQuest | (older* OR elder* OR senior* OR geriatric OR aged OR “late life” OR “late onset” OR “old age” ) AND ( lonel* OR "social isolation" ) AND ( promot* OR prevent* OR support OR program* OR educat* OR reduc* OR minim* OR less* OR therap* OR interven* OR treat* ) AND ( review OR overview OR evaluat* OR discuss* OR “systematic review” or “meta-analysis” or “research synthesis” or “research integration” or “information synthesis” or “data synthesis” or “data extraction”) | 1970 to present | 149 |
| Google Scholar | allintitle: ("social isolation" OR loneliness) AND (review OR meta-analysis OR overview) AND (elderly OR older OR aging OR geriatric) | All time | 28 |
| JBI Database of Systematic Reviews and Implementation Reports via Joanna Briggs Institute | older* OR elder* OR senior* OR geriatric OR aged OR “late life” OR “late onset” OR “old age”; lonel* OR "social isolation"; promot* OR prevent* OR support OR program* OR educat* OR reduc* OR minim* OR less* OR therap* OR interven* OR treat*; review OR overview OR evaluat* OR discuss* OR “systematic review” or “meta analysis” or “research synthesis” or “research integration” or “information synthesis” or “data synthesis” or “data extraction” | All time | 19 |
| PsycInfo via EBSCOhost | Any Field: lonel* *OR* Any Field: "social isolation" *AND* (Any Field: promot* *OR* Any Field: prevent* *OR* Any Field: support*OR* Any Field: program* *OR* Any Field: educat* *OR* Any Field: reduc* *OR* Any Field: minim* *OR* Any Field: less* *OR* Any Field: therap**OR* Any Field: interven* *OR* Any Field: treat*) *AND* Any Field: (review OR overview OR evaluation OR discussion OR "systematic review" OR "meta-analysis" OR "research synthesis" OR “research integration” OR “information synthesis” OR “data synthesis” OR “data extraction”) *AND* Age Group: Aged (65 yrs & older) | 1970 to present | 550 |
| MEDLINE via PubMed | ((((review OR overview OR evaluat* OR discuss* OR "Review" [Publication Type] OR "Meta-Analysis" [Publication Type] OR “systematic review” or “meta-analysis” or “research synthesis” or “research integration” or “information synthesis” or “data synthesis” or “data extraction”)) AND ((promot* OR prevent* OR support OR program* OR educat* OR reduc* OR minim* OR less* OR therap* OR interven* OR treat*))) AND ((lonel* OR social isolat* OR "social isolation" OR "Social Isolation"[Mesh] OR "Loneliness"[Mesh]))) AND ((older* OR elder* OR senior* OR geriatric OR aged OR “late life” OR “late onset” OR “old age” OR "aged"[Mesh])) | 1970 to present | 2318 |
| Web of Science Core Collection | TS=(older* OR elder* OR senior* OR geriatric OR aged OR late life OR late onset OR "old age" ) AND TS=( lonel* OR "social isolation" ) AND TS=( promot* OR prevent* OR support OR program* OR educat* OR reduc* OR minim* OR less* OR therap* OR interven* OR treat* ) AND TS=( review OR overview OR evaluat* OR discuss* OR systematic review or meta-analysis or research synthesis or research integration or information synthesis or data synthesis or data extraction ) | 1980 to present | 1614 |
